# Microbial Hydrolysis of Racemic β-Aryl-γ-ethylidene-γ-lactones and Antifeedant Activity of the Products against *Alphitobius diaperinus* Panzer

**DOI:** 10.3390/molecules23071516

**Published:** 2018-06-23

**Authors:** Andrzej Skrobiszewski, Witold Gładkowski, Marcelina Mazur, Maryla Szczepanik, Gabriela Maciejewska, Czesław Wawrzeńczyk

**Affiliations:** 1 Department of Chemistry, Wroclaw University of Environmental and Life Sciences, 50-375 Wrocław, Poland; glado@poczta.fm (W.G.); marcelina.mazur@up.wroc.pl (M.M.); czeslaw.wawrzenczyk@up.wroc.pl (C.W.); 2 Department of Invertebrate Zoology, Nicolaus Copernicus University, 87-100 Toruń, Poland; mszczep@umk.pl; 3 Central Laboratory of the Instrumental Analysis, Wroclaw University of Technology, 50-370 Wroclaw, Poland; gabriela.maciejewska@pwr.edu.pl

**Keywords:** unsaturated lactones, kinetic resolution, microbial hydrolysis, antifeedant activity

## Abstract

Hydrolysis of (±)-β-aryl-γ-ethylidene-γ-lactones by fungal strain *Aspergillus ochraceus* AM370 afforded (−)-(*S*)-γ-ethylidene-γ-lactones **2a**–**d** and (+)-(*R*)-γ-ketoacids **3a**–**d**. Enantiomeric purity of the unreacted lactones was strictly related to a size of an aryl substituent at C-4 of γ-lactone ring, with the highest *ee* (77%) obtained for the (−)-(*S*)-γ-ethylidene-γ-lactone possessing unsubstituted benzene ring (**2a**) and the lowest one (15%) determined for the (−)-(*S*)-γ-ethylidene-γ-lactone with bulky 1,3-benzodioxole system (**2d**). Lactones **2a**–**d**, both racemic and enantiomerically enriched, as well as products of their hydrolysis showed varying degrees of feeding deterrent activity against lesser mealworm, *Alphitobius diaperinus* Panzer, which depended on the structure of the compound and the developmental stage of the lesser mealworm. In the case of adults, more active were γ-lactones **2a**–**d**, compared with ketoacids **3a**–**d**. Only in the case of lactone **2a** was the effect of configuration of stereogenic center on the activity found. Particularly strong deterrents against this stage (T > 180) were racemic and (−)-(*S*)-γ-ethylidene-γ-lactone with *p*-methoxysubstituted phenyl ring (**2c**).

## 1. Introduction

One of the most common methods for the production of enantiopure compounds is the enzymatic kinetic resolution of racemic mixtures [1,2,3,4]. In this process, the yield of products is limited to a maximum of 50% due to the fact that one of enantiomers remains unreacted [3,4]. On the contrary, in dynamic kinetic resolution, the non-reacting enantiomer is racemized in situ, which allows to overcome the limitation of 50% conversion [5,6]. Despite these limitations, kinetic resolution is still a valuable tool leading to optically pure compounds. In enzymatic kinetic resolution, oxidoreductases [7] and hydrolases are mainly used [8]. Among hydrolases, lipases found a widest application due to a high substrate specificity, selectivity, stability, ease of immobilization, and no need of cofactors [9,10,11].

In the last few years, our scientific interests have concentrated on the synthesis of compounds with lactone motif because of wide spectrum of their biological activity such as antifeedant [12,13], antifungal [14,15], antibacterial [16], antiviral [17], anti-inflammatory [18], antioxidant [19,20] and anticancer [21,22]. Observed dependence of these properties on the configuration of chiral centers, as well as the application of optically active γ-lactones as chiral building blocks in the synthesis of natural products [23,24,25] caused the development of new methods for the production of enantiomerically enriched γ-lactones. One of them is a microbial enantioselective hydrolysis of racemic mixtures of γ-lactones, in which lactonohydrolases (esterase-family enzymes) are involved. The main two sources of lactonohydrolases are fungi from the genera *Fusarium*, *Aspergillus*, *Volutella*, *Gibberella* and bacteria belonging to the genera *Achromobacter*, *Bacillus*, *Agrobacterium*, *Acinetobacter* [26,27]. Among fungal lactonohydrolases the best characterized was that from *Fusarium oxysporum* and among bacterial lactonohydrolases the one from *Agrobacterium tumefaciens* [27]. Fungal lactonohydrolases were widely applied to kinetic resolution of dl-pantolactone, which is an important intermediate for the production of *d*-pantothenic acid [28,29]. Enantiomerically enriched γ-lactones were also obtained by lipase-mediated kinetic resolution of their racemic mixtures [30,31].

Recently, we have taken up research on synthesis of a series of β-aryl-γ-lactones and evaluation of their biological activity. Since then we have proved their antifeedant, antifungal, antibacterial, and anticancer activity [32,33,34,35,36,37]. We have also reported convenient routes to optically active β-aryl-δ-iodo-γ-lactones [35,36] and β-aryl-δ-hydroxy-γ-lactones [37], in which a key step is lipase-catalyzed kinetic resolution of allylic alcohols. Some time ago, we also developed the method of kinetic resolution of β-methyl-γ-ethylidene lactones using lipases [31]. In this paper, we would like to present the results of the hydrolysis of β-aryl-γ-ethylidene-γ-lactones by fungi and evaluation of the antifeedant activity of racemic lactones, as well as products of their hydrolysis against a cosmopolitan pest, lesser mealworm *Alphitobius diaperinus* Panzer occurring mainly in broiler grow-out houses.

## 2. Results and Discussion

### 2.1. Hydrolysis of Lactones ***2a**–**d***

**Scheme 1:**
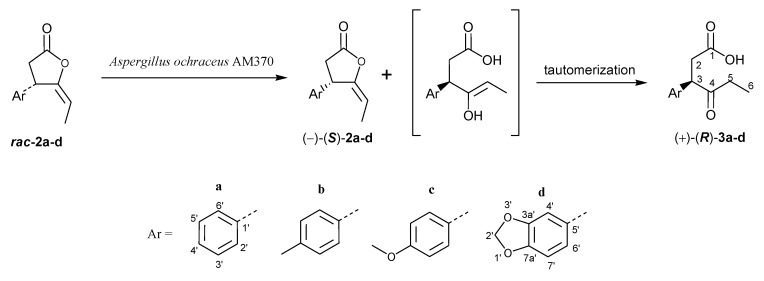
Enantioselective hydrolysis of (±)-γ-ethylidene-γ-lactones **2a**–**d**. *Reagents and conditions:* (**a**) *Aspergillus ochraceus* AM 370, 3 days for **2a**,**b** and 5 days for **2c**,**d**.

In the screening tests, racemic γ-ethylidene-γ-lactone possessing unsubstituted benzene ring (**2a**) was used as a model substrate. Among six fungal strains tested, only *Aspergillus ochraceus* AM370 showed hydrolytic activity towards the lactone **2a**. This strain was previously found to catalyze the hydrolysis of 1-phenylethyl acetate and subsequent oxidation of 1-phenylethanol to acetophenone [38]. In the multiplied scale biotransformations, besides the model substrate **2a**, its analogues with *p*-methylphenyl (**2b**), *p*-methoxyphenyl (**2c**) and 1,3-benzodioxole substituents (**2d**) were subjected to transformation. Hydrolysis afforded corresponding (+)-γ-ketoacids: three new (**3b**–**d**) and one known (**3a**) [39] as well as unreacted, optically active (−)-lactones **2a**–**d** (Scheme 1).

Structures of (+)-γ-ketoacids **3a**–**d** were confirmed by spectroscopic data. The presence of a carboxyl group was proved by broad bands of O-H stretching vibrations at the range of 3519–2930 cm^−1^ for **3a**, 3424–2926 cm^−1^ for **3b**, 3367–2930 cm^−1^ for **3c**, 3424–2360 for **3d** and strong bands of C=O stretching vibrations at 1713, 1715, 1769, 1728 cm^−1^ for **3a**–**d**, respectively. In the ^13^C NMR spectra signals (δ = 177.6 for **3a**, 177.2 for **3b**, 177.6 for **3c** and 177.2 ppm for **3d**) undoubtedly indicated the presence of carbon atom from carboxylic group. Signals from C-4 for **3a**–**d** were found at δ = 209.7, 209.7, 209.8 and 209.5, respectively and confirmed the presence of carbonyl group. In the ^1^H NMR spectra characteristic shapes and chemical shifts of signals from protons of ethyl group (e.g., for ketoacid **3b** triplet at 0.97 ppm and quartet at 2.42 ppm) proved the location of carbonyl group in these molecules at C-4. Formation of ketoacid is the result of keto-enol tautomerism and predomination of keto form over an enol tautomer which is a direct product of γ-ethylidene-γ-lactone hydrolysis (Scheme 1).

**Figure 1:**
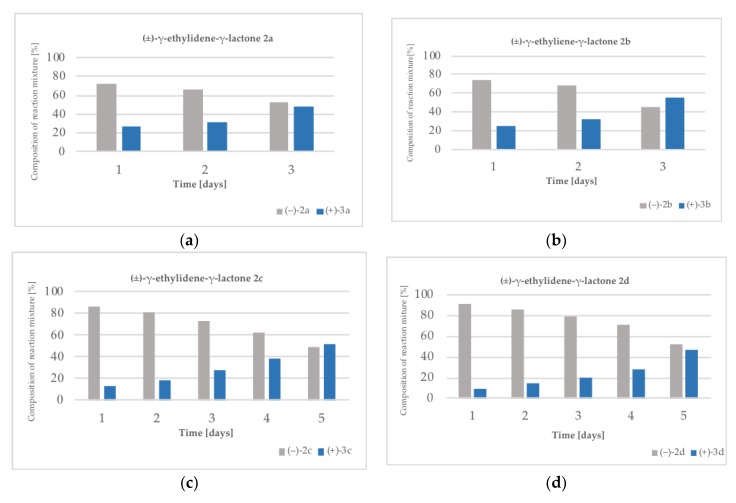
Time course of the hydrolysis of (±)-γ-ethylidene-γ-lactones **2a**–**d** in the culture of *Aspergillus ochraceus* AM370.

Biotransformation rate was strictly related to the size of aryl substituent. After 24 h for γ-lactone with unsubstituted benzene ring **2a** (Figure 1a) and methyl substituted benzene ring **2b** (Figure 1b) conversion exceeded 25%. Significantly slower reaction rate was observed for lactone with methoxy substituted benzene ring (**2c**) (Figure 1c) and 1,3-benzodioxole system (**2d**) (Figure 1d); for these substrates during first 24 h conversion reached only 13% and 9%, respectively. After three days of process conversion of lactones, **2a** and **2b** stopped at approximately 50%; at the same time, conversion of lactones **2c** and **2d** was only 28% and 21%, respectively. For these two substrates only after five days of biotransformation 51% conversion in the case of **2c** and 48% in the case of **2d** was observed and no further transformation was observed. In order to achieve maximal isolated yield of products with highest possible optical purity, biotransformations were stopped after three days in the case of lactones **2a**,**b** and five days in the case of lactones **2c**,**d**.

Enantiomeric excesses of unreacted isomers of lactones **2a**–**d** determined at about 50% conversion are given in Table 1, which clearly shows a decrease of enantiomeric purity of slower reacting lactones along with the increase of size of an aryl substituent. The highest *ee* was achieved for γ-lactone possessing unsubstituted benzene ring (**2a**). Lactone **2b** with methyl substituted benzene ring was obtained with 56% *ee*, the presence of methoxy group (compound **2c**) resulted in lowering the optical purity of unreacted lactone. The lowest enantioselectivity of reaction was observed for the lactone with bulky 1,3-benzodioxole system **2d**. The explanation of decreasing enantiomeric purity of lactones **2a**–**d** is probably a steric hindrance which impedes an access of the substrate to the pocket of enzyme. Similar effect of 1,3-benzodioxole system on the effectiveness of the kinetic resolution was observed in our earlier studies over the lipase-catalyzed transesterification of (*E*)-4-(benzo[d][1′,3′]dioxol-5′-yl)-but-3-en-2-ol [40]. Resolution of bulky substrates has been a remarkable challenge in organic chemistry. These limitations can be overcome by mutagenesis, the example is engineering of an epoxide hydrolase from *Bacillus megaterium* ECU1001 to improve resolution of α-naphtyl glycidyl ether, bulky precursor of (*S*)-propranolol-drug blocking β-adrenergic receptor [41].

**Scheme 2:**
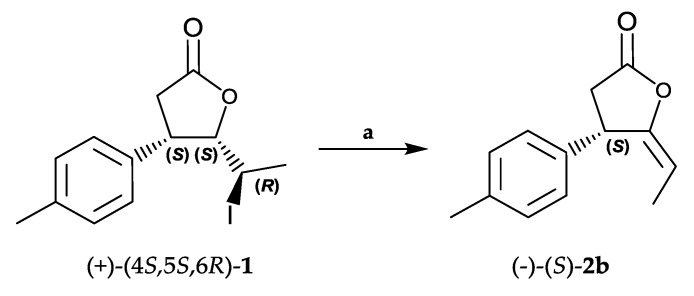
Dehydrohalogenation of (+)-*cis*-(*4S*,*5S*,*6R*)-δ-iodo-γ-lactone 1. *Reagents and conditions:* (**a**) 1,8-diazabicyclo[5.4.0]undec-7-ene, benzene, 80 °C, 6.5 h.

Enantiopreference of *Aspergillus ochraceus* AM370 during hydrolysis of racemic γ-ethylidene-γ-lactones **2a**–**d** was proved based on the determination of configuration of slowly reacting enantiomer of the γ-lactone with *p*-methylphenyl substituent (−)-**2b**. For that purpose, optically pure (−)-γ-ethylidene-γ-lactone **2b** with *S*-configuration at C-4 was obtained from corresponding chiral δ-iodo-γ-lactone **1** with defined configuration of chiral centers [35] by dehydrohalogenation with DBU (Scheme 2). Negative specific rotation sign measured for this isomer and for enantiomer of **2b**, and slower hydrolyzation by *Aspergillus ochraceus* AM370 (Scheme 1) proved the *S* configuration of the letter. Taking into consideration the structural similarity of all four lactones subjected to microbial hydrolysis, the *S* configuration was also assigned to the slower reacting (−)-enantiomers of γ-lactones **2a**, **2c** and **2d**. Consequently, we could conclude that *Aspergillus ochraceus* AM370 prefers hydrolysis of (*R*-lactones **2a**–**d** to corresponding (*R*)-ketoacids **3a**–**d**. (Scheme 1). Unfortunately, despite using different GC and HPLC chiral columns and separation conditions, including resolution of methyl and ethyl ester derivatives, our attempts to separate enantiomers of **3a**–**d** chromatographically failed and their enantiomeric compositions could not be determined.

The obtained results showed the effect of aryl substituent at C-4 on the rate of hydrolysis of γ-lactone ring and enantiomeric purity of the unreacted lactone. In the earlier work, Enzelberger et al. [30] conducted hydrolysis of a series of γ-lactones catalyzed by lipase from *Pseudomonas* sp. This enzyme preferentially hydrolyzed (*R*)-enantiomers of γ-lactones which is coherent with our results and preference of *Aspergillus ochraceus* AM370 to the hydrolysis of (*R*)*-*γ-lactones **2a**–**d**. Commercially available enzymes were also used for the hydrolysis of β-methyl-γ-ethylidene lactones by Fajkowska et al. [31]. Lipase PS (from *Pseudomonas cepacia*) showed moderate enantiopreference to (+)-isomers of γ-lactones leaving unreacted (−)-isomers with *ee* in the range of 18–60% depending on the reaction time (4–15 days). The change of enantiopreference was observed if the reaction was catalyzed by lipase from *Rhizopus niveus*. In this case (−)-isomers of unsaturated γ-lactones were hydrolyzed faster to the corresponding ketoacids.

### 2.2. Antifeedant Activity

In our previous studies we presented *i.a.* synthesis of racemic γ-ethylidene-γ-lactones with aryl substituents at β-position (**2a**–**d**) and evaluation on their antifeedant activity towards *Tribolium confusum*, *Trogoderma granarium* and *Sitophilus granaries* [33,34]. In this work, we tested both racemic γ-ethylidene-γ-lactones and products of their hydrolysis for the antifeedant activity against lesser mealworm *Alphitobius diaperinus* Panzer (Table 2). The comparative analysis of racemic and optically active form of lactones was supposed to deliver the information about the possible relationship between configuration of these compounds and their antifeedant activity. On the other hand, for the first time we could also compare the antifeedant activity of γ-lactones **2a**–**d** with ketoacids **3a**–**d**, products of their metabolism in the culture of *Aspergillus ochraceus* AM370. Nevertheless, the influence of aryl substituent could also be determined.

Feeding deterrent activity of compounds studied depended on their structure and the developmental stage of the lesser mealworm. Among racemic lactones particularly high activity against adults (T = 185.52) was observed for lactone with methoxysubstituted benzene ring (**2c**) (Entry 3). Very good antifeedants, with T coefficient higher than 150 for both developmental stages, were lactones with methylsubstituted benzene ring (**2b**) and 1,3-benzodioxole system (**2d**) (Entry 2,4). The relatively weakest feeding deterrent among racemic lactones, especially against larvae, was γ-lactone with unsubstituted benzene ring (**2a**) (Entry 1). Thus, the racemic lactones containing additional substituents at the benzene ring are stronger feeding deterrents and this relationship is particularly pronounced in the case of larvae of *A. diaperinus*. Similar trend was observed among enantiomerically enriched γ-lactones (Entry 5–8). However, in this series of compounds the activity was higher against adults of *A. diaperinus* in comparison with larvae. Feeding of adults was reduced in highest extent by (−)-(*S*)-γ-ethylidene-γ-lactone with methoxy group (−)-**2c** (Entry 7). Interestingly, the high values of T coefficients for this lactone (T = 188.75) and its racemic form (Entry 3) was the most similar to azadirachtin, the most active antifeedant of natural origin. Among enantiomerically enriched lactones (−)-(*S*)-γ-ethylidene-γ-lactone with methoxy group exhibited also the highest activity against larvae of the lesser mealworm (T = 140.95). These results confirmed significant positive influence of this substituent on the deterrent activity towards the lesser mealworm which was also observed for the series of β-aryl-γ-lactones in the previous tests against storage pests [33,34]. The presence of 1,3-benzodioxole system caused only a slight decrease of activity but these differences were not statistically significant (Entry 8).

Only in the case of lactones with unsubstituted benzene ring (**2a**) a significant relationship between their configuration and antifeedant activity was observed. For adults, more active was racemic lactone **2a** (Entry 1) than its (−)-(*S*)-enantiomer (Entry 5); for larvae this relationship was reversed. Therefore, it can be supposed that in this case (*S*)-enantiomer is more active against larvae and (*R*)-enantiomer is better antifeedant against adults of *A. diaperinus*. The comparison of the other three pairs of racemic lactones and their (−)-(*S*)-enantiomers indicates no significant differences in their activity; probably because of lower enantiomeric excesses of (−)-(*S*)-enantiomers **2c**–**d** comparing to lactone (−)-(*S*)-**2a**.

Ketoacids **3a**–**d** differed significantly from lactones in terms of the deterrent activity and they were weaker antifeedants for adults than for larvae of the lesser mealworm. In the case of adults decrease in activity along with the increase of size of an aryl substituent was observed. Ketoacid (+)-(*R*)-**3a** with unsubstituted benzene ring strongly reduced feeding of adults, but only in the choice test. In the no choice tests the level of feeding in the presence of all ketoacids was at a similar level. Comparing to lactones, ketoacids **3a**–**d** were definitely weaker antifeedants against adults, with the exception of compounds (+)-(*R*)-**3a** (Entry 9) whose activity was similar to (−)-(*S*)-γ-lactone **2a** (Entry 5). In the case of larvae, ketoacid (+)-(*R*)-**3a** with unsubstituted benzene ring (**2a**) was more active than its parent lactone whereas the activities of ketoacids with substituted benzene ring (**3b**,**d**) (Entry 10–12) were lower than those of corresponding racemic substrates (±)-**2b**,**d**.

## 3. Materials and Methods

### 3.1. Analysis

The course of transformations was monitored by thin layer chromatography (TLC) on silica gel-coated aluminum plates (DC-Alufolien Kieselgel 60 F_254_, Merck, Darmstadt, Germany). Composition of products mixture was determined by gas chromatography (GC) on Agilent Technologies 6890N (Palo Alto, CA, USA) instrument using a DB-5HT column (polyimide-coated fused silica tubing, 30 m × 0.25 mm × 0.10 μm) with hydrogen as gas carrier and autosampler. Following temperature program was applied: injector 220 °C, detector (FID) 330 °C, initial temperature column 90 °C, 90–330 °C (rate 20 °C·min^−1^), final temperature column 330 °C (hold 2 min).

Enantiomeric composition of optically active lactones was determined by chiral gas chromatography (CGC) using a CP-Chirasil-Dex CB chiral column (25 m × 0.25 mm × 0.25 μm). The temperature program was as follows: injector 260 °C, detector (FID) 280 °C, initial column temperature 60 °C, 60–200 °C (rate 2 °C/min), final temperature column 200 °C (hold 1 min). Products of biotransformation were separated by preparative column chromatography on silica gel (Kieselgel 60, 230–400 mesh, Merck).

^1^H NMR, ^13^C NMR, HMBC and HMQC spectra were recorded in CDCl_3_ solutions on a Bruker Avance DRX 300 spectrometer (Rheinstetten, Germany). IR spectra were determined using a Mattson IR 300 Thermo Nicolet spectrophotometer. High resolution mass spectra (HRMS) were recorded using electron spray ionization (ESI) technique on Waters ESI-QTOF Premier XE spectrometer. The optical rotations were measured on a Jasco P-2000-Na digital polarimeter with an intelligent Remote Module (iRM) controller. The melting points (uncorrected) were determined on Boetius apparatus.

### 3.2. Chemicals

Racemic (*E*)-5-ethylidene-4-phenyldihydrofuran-2-one (**2a**), (*E*)-5-ethylidene-4-(4′ methylphenyl)dihydrofuran-2-one (**2b**), (*E*)-5-ethylidene-4-(4′-methoxyphenyl)dihydrofuran-2-one (**2c**) and (*E*)-4-(benzo[*d*][1,′3′]dioxol-5′-yl)-5-ethylidenedihydrofuran-2-one (**2d**) were synthesized previously by our research team from corresponding aromatic aldehydes [33,34]. (+)-*cis*-(4*S*,5*S*,6*R*)-5-(1-Iodoethyl)-4-(4′-methylphenyl)dihydrofuran-2-one (1) was synthesized earlier from (+)-(2*R*,3*E*)-4-(4′-methylphenyl)but-3-en-2-ol [35]. 1,8-diazabicyclo[5.4.0]undec-7-ene (DBU, purity ≥ 98%) was purchased form Merck. Sodium chloride, anhydrous magnesium sulphate, hydrochloric acid (35–37%) and organic solvents used for the synthesis and extraction were purchased form Chempur. The chemicals used for the preparation of a growing media were purchased from BTL.

### 3.3. Synthesis of (−)-(S,E)-5-Ethylidene-4-(4′methylphenyl)dihydrofuran-2-one *[(−)-**2b**]*

A mixture of (+)-δ-iodo-γ-lactone 1 (1.22 g, 3.7 mmol) and 1.8-diazabicyclo[5.4.0]undec-7-ene (DBU) (1.5 mL, 10 mmol) was dissolved in anhydrous benzene (20 mL) and heated under reflux for 6.5 h. The solvent was evaporated in vacuo and then the crude reaction mixture was acidified with 1M HCl. The products were extracted with diethyl ether (4 × 30 mL). The combined extracts were washed with brine, dried over anhydrous MgSO_4_ and filtered. The organic solvent was evaporated and (−)-(*S*,*E*)-5-ethylidene-4-(4′-methylphenyl)dihydrofuran-2-one (**2b**) was isolated as the major product by column chromatography (hexane/acetone, 10:1, *v*/*v*) [Yield 48% (0.36 g), *ee* = 97%, ${\left[\alpha \right]}_{D}^{20}$ = −111.0 (*c* 0.5, CH_2_Cl_2_)]. Its spectroscopic data were in accordance with these reported earlier for racemic lactone **2b** [33].

### 3.4. Microorganism and Growing Medium

Fungal strains: *Beauveria bassiana* AM278, *Aspergillus ochraceus* AM370, *Fusarium avenaceum* AM12, *Absidia cylindrospora* AM336, *Cladosporium avellaneum* AM135 and *Penicillium frequentans* AM359 were from the Collection of Department of Chemistry Wroclaw University of Environmental and Life Sciences. The strains were cultivated at 28 °C on Sabouraud agar slants consisting of aminobac (5 g), peptone K (5 g), glucose (40 g) and agar (15 g) dissolved in water (1 L) at pH 5.5 and stored in refrigerator at 4 °C.

### 3.5. Screening Procedure

Fungal strains were cultivated in 250 mL Erlenmeyer flasks containing 50 mL of the culture medium (3% glucose and 1% peptobac, pH = 6) at room temperature from 3 to 7 days. Racemic (*E*)-5-ethylidene-4-phenyldihydrofuran-2-one (**2a**) (10 mg/1 mL of acetone) was added to 300 mL Erlenmeyer flask with grown fungal biomass. Biotransformation was carried out with continuous shaking (140 rpm) at room temperature. Samples were taken after 1, 2, 3, 4, and 5 days of transformation, products were extracted with methylene chloride (3 × 10 mL). After centrifugation (7000 rpm) organic layers were separated, combined, dried over anhydrous MgSO_4_ and filtered. After evaporation of solvent in vacuo the crude mixture was analyzed by TLC and GC. In order to identify the metabolites of the tested microorganisms, fungal cultures without addition of the substrate **2a** were cultivated in the same conditions. The stability of the model substrate **2a** in the culture media was checked as well.

### 3.6. General Procedure for Multiplied Scale Biotransformation of Racemic γ-Lactones ***2a**–**d*** and Isolation of the Products

*Aspergillus ochraceus* AM370 strains was cultivated in 16 Erlenmayer flasks (250 mL volume) containing 50 mL of biotransformation medium described in screening procedure (Section 3.5). To each of 16 Erlenmeyer flasks containing biomass of *Aspergillus ochraceus* AM370, 10 mg of substrate dissolved in 1 mL of acetone was added (total volume of biotransformation medium was 800 mL, total substrate amount 160 mg). All reaction conditions were the same as described in the screening procedure. The biotransformations were carried out with continuous shaking (140 rpm) at room temperature. The progress of transformations was followed by GC and TLC (chloroform/methanol, 15:1, *v*/*v*) for visualization of γ-lactones (−)-**2a**–**d** and (chloroform/methanol/formic acid, 10:1:0.5, *v*/*v*) for detection of (+)-(*R*)-γ-ketoacids **3a**–**d**. After 3 days (in the case of lactones **2a**,**b**) or 5 days (in the case of lactones **2c**,**d**) biomass was centrifuged and the products were extracted from the liquid medium with methylene chloride (3 × 30 mL). Extracts were dried over anhydrous MgSO_4_ and filtered. Solvent was evaporated in vacuo and the biotransformation products were separated by column chromatography using two different developing systems: (chloroform/methanol, 15:1, *v*/*v*) to elute (−)-(*S*)-γ-ethylidene-γ-lactones **2a**–**d**, followed by (chloroform/methanol/formic acid, 10:1:0.5, *v*/*v*) to isolate (+)-(*R*)-γ-ketoacids **3a**–**d**.

### 3.7. Biotransformation of (±)-(E)-5-Ethylidene-4-phenyldihydrofuran-2-one (***2a***)

Transformation of (±)-lactone **2a** (160 mg, 0.85 mmol) afforded mixture of (−)-**2a** and (+)-**3a**. Their physical and spectral data are shown below:

*(−)-(S*,*E)-5-Ethylidene-4-phenyldihydrofuran-2-one* (−)-**2a**. Yield 18% (0.03 g), *t*_R_ = 4.903, *ee* = 77%, ${\left[\alpha \right]}_{D}^{20}$ = −74.8 (*c* 0.3, CH_2_Cl_2_); spectroscopic data in accordance with these reported for its racemic form [33].

*(+)-(R)-4-Oxo-3-phenylhexanoic acid* (+)-**3a**. Yield 13% (0.023 g); oil, *t*_R_ = 5.046, ${\left[\alpha \right]}_{D}^{20}$ = +4.8 (*c* 0.3, CH_2_Cl_2_), IR (film, cm^−1^): 3519–2930 (s, b), 1713 (s), 1455 (s), 1239 (m), 756 (s), 702 (s); ^1^H NMR (300 MHz, CDCl_3_) δ: 0.97 (t, *J* = 7.3 Hz, 3H, CH_3_-6), 2.43 (m, 2H, CH_2_-5), 2.57 (dd, *J* = 17.4 and 4.8 Hz 1H, one of CH_2_-2), 3.29 (dd, *J* = 17.4 and 10.0 Hz, 1H, one of CH_2_-2), 4.15 (dd, *J* = 10.0 and 4.8 Hz, 1H, H-3), 7.18–7.36 (two m, 5H, aromatic protons); ^13^C NMR (75 MHz, CDCl_3_) δ: 7.8 (C-6), 34.6 (C-5), 36.8 (C-2), 53.6 (C-3), 127.8 (C-4′), 128.2 (C-2′ and C-6′) 129.2 (C-3′ and C-5′), 137.5 (C-1′), 177.6 (C-1), 209.7 (C-4); HRMS (ESI): *m*/*z* calcd. for C_12_H_14_O_3_ [M + H]^+^: 205.0865, found 205.0867.

### 3.8. Biotransformation of (±)-(E)-5-Ethylidene-4-(4′-methylphenyl)dihydrofuran-2-one (***2b***)

Transformation of (±)-lactone **2b** (160 mg, 0.79 mmol) afforded mixture of (−)-**2b** and (+)-**3b**. Their physical and spectral data are as follows:

*(−)-(S*,*E)-5-Ethylidene-4-(4′-methylphenyl)dihydrofuran-2-one* (−)*-***2b**. Yield 17% (0.04 g), *t*_R_ = 5.358, *ee* = 56%, ${\left[\alpha \right]}_{D}^{20}$ = −13.6 (*c* 0.8, CH_2_Cl_2_); spectroscopic data in accordance with these reported for its racemic form [33].

*(+)-(R)-3-(4′-Methylphenyl)-4-oxohexanoic acid* (+)-**3b**. Yield 12% (0.021 g); oil, *t*_R_ = 5.711, ${\left[\alpha \right]}_{D}^{20}$ = +98.1 (*c* 0.2, CH_2_Cl_2_), IR (film, cm^−1^): 3424–2926 (s, b), 1715 (s), 1438 (s), 1252 (m), 834 (s), 737 (s); ^1^H NMR (300 MHz, CDCl_3_) δ: 0.97 (t, *J* = 7.3 Hz, 3H, CH_3_-6), 2.32 (s, 3H, Ph-CH_3_), 2.42 (q, *J* = 7.3 Hz, 2H, CH_2_-5), 2.55 (dd, *J* = 17.3 and 4.8 Hz 1H, one of CH_2_-2), 3.26 (dd, *J* = 17.3 and 9.8 Hz, 1H, one of CH_2_-2), 4.11 (dd, *J* = 9.8 and 4.8 Hz, 1H, H-3), 7.06–7.15 (two m, 4H, *p*-C_6_H_4_); ^13^C NMR (75 MHz, CDCl_3_) δ: 7.8 (C-6), 21.1 (Ph-CH_3_), 34.5 (C-5), 36.8 (C-2), 53.3 (C-3), 128.1 (C-2′ and C-6′), 129.1 (C-3′ and C-5′), 134.4 and 137.5 (C-1′ and C-4′), 177.2 (C-1), 209.7 (C-4); HRMS (ESI): *m*/*z* calcd. for C_13_H_16_O_3_ [M + H]^+^: 219.1021, found 219.1013.

### 3.9. Biotransformation of (±)-(E)-5-Ethylidene-4-(4′-methoxyphenyl)dihydrofuran-2-one (***2c***)

Transformation of (±)-lactone **2c** (160 mg, 0.73 mmol) afforded mixture of (−)-**2c** and (+)-**3c**. Their physical and spectral data are given below.

*(−)-(S*,*E)-5-ethylidene-4-(4′-methoxyphenyl)dihydrofuran-2-one* (−)-**2c**. Yield 14% (0.02 g), *t*_R_ = 5.981, *ee* = 22%, ${\left[\alpha \right]}_{D}^{20}$ = −15.4 (*c* 0.2, CH_2_Cl_2_); spectroscopic data are consistent with these reported for its racemic form [33].

*(+)-(R)-3-(4′-methoxyphenyl)-4-oxohexanoic acid* (+)-**3c**. Yield 12% (0.02 g); oil, *t*_R_ = 6.144, ${\left[\alpha \right]}_{D}^{20}$ = +45.8 (*c* 0.1, CH_2_Cl_2_), IR (film, cm^−1^): 3367–2930 (s, b), 1769 (s), 1511 (m), 1265 (s), 744 (m); ^1^H NMR (300 MHz, CDCl_3_) δ: 0.96 (t, *J* = 7.3 Hz, 3H, CH_3_-6), 2.41 (q, *J* = 7.3 Hz, 2H, CH_2_-5), 2.54 (dd, *J* = 17.3 and 4.9 Hz 1H, one of CH_2_-2), 3.21 (dd, *J* = 17.3 and 9.8 Hz, 1H, one of CH_2_-2), 3.78 (s, 3H, -OCH_3_), 4.08 (dd, *J* = 9.8 and 4.9 Hz, 1H, H-3), 6.82-6.87 (m, 2H, H-3′ and H-5′), 7.08–7.13 (m, 2H, H-2′ and H-6′); ^13^C NMR (75 MHz, CDCl_3_) δ: 7.9 (C-6), 34.5 (C-5), 36.9 (C-2), 52.8 (C-3), 55.3 (-OCH_3_), 114.6 (C-3′ and C-5′), 129.3 (C-2′ and C-6′), 129.4 (C-1′), 159.1 (C-4′), 177.6 (C-1), 209.8 (C-4); HRMS (ESI): *m*/*z* calcd. for C_13_H_16_O_4_ [M + H]^+^: 235.0970, found 235.0980.

### 3.10. Biotransformation of (±)-(E)-4-(benzo[d][1,′3′]dioxol-5′-yl)-5-ethylidenedihydrofuran-2 one (***2d***)

Transformation of (±)-lactone **2d** (160 mg, 0.69 mmol) afforded mixture of (−)-**2a** and (+)-**3a**. Their physical and spectral data are shown below:

*(−)-(S*,*E)-4-(Benzo[d][1*,*′3′]dioxol-5′-yl)-5-ethylidenedihydrofuran-2 one* (−)-2d. Yield 12% (0.02 g), *t*_R_ = 6.434, *ee* = 15%, ${\left[\alpha \right]}_{D}^{20}$ = −11.4 (*c* 0.1, CH_2_Cl_2_), spectroscopic data in accordance with these reported for its racemic form [33].

*(+)-(R)-3-(Benzo[d][1*,*′3′]dioxol-5′-yl)-4-oxohexanoic acid* (+)-3d. Yield 12% (0.02 g); oil, *t*_R_ = 6.647, ${\left[\alpha \right]}_{D}^{20}$ = +25.0 (*c* 0.1, CH_2_Cl_2_), IR (film, cm^−1^): 3424–2360 (s, b), 1728 (s), 1513 (m), 1241 (s), 828 (s), 719 (s); ^1^H NMR (300 MHz, CDCl_3_) δ: 0.98 (t, *J* = 7.2 Hz, 3H, CH_3_-6), 2.44 (m, 2H, CH_2_-5), 2.54 (dd, *J* = 17.3 and 4.9 Hz 1H, one of CH_2_-2), 3.21 (dd, *J* = 17.3 and 9.8 Hz, 1H, one of CH_2_-2), 4.05 (dd, *J* = 9.8 and 4.9 Hz, 1H, H-3), 5.95 (s, 2H, CH_2_-2′), 6.64 (dd, *J* = 8.7 and 1.8 Hz, 1H, H-6′), 6.67 (d, *J* = 1.8, 1H, H-4′), 6.78 (d, *J* = 8.7 Hz, 1H, H-7′); ^13^C NMR (75 MHz, CDCl_3_) δ: 7.9 (C-6), 34.5 (C-5), 36.9 (C-2), 53.2 (C-3), 101.2 (C-2′), 108.3 (C-4′), 108.8 (C-7′), 121.7 (C-6′), 131.1 (C-5′), 147.2 (C-3a′), 148.2 (C-7a′), 177.2 (C-1), 209.5 (C-4); HRMS (ESI): *m*/*z* calcd. for C_13_H_14_O_5_ [M + H]^+^: 249.0763, found 249.0774.

### 3.11. Antifeedant Activity Assays

Feeding deterrent activity of (±)-γ-ethylidene-γ-lactones **2a**–**d** and products of their biotransformations (−)-**2a**–**d** and (+)-**3a**–**d** against *Alphitobius diaperinus* Panzer in the choice test and the no-choice test was evaluated. Oat flakes were used as the test food. For feeding assays acetone solutions of tested compounds at a concentration of 10 mg/10 mL^−1^ were prepared. A quantity of 1 mL of a test solution or acetone alone as control was applied to 1 g of flakes by micropipette. After evaporation of solvent (30 min of air drying), the flakes were weighted and placed in petri dishes (15 cm diameter) together with ten larvae (25–30 days old) and ten unsexed adults (7–10 days old). In order to obtain large number of insects at approximately same age, the Rice-Lambkin culture method for lesser mealworm was used.

In the choice tests (where insects could choose either control or treated food), control and treated flakes in petri dishes were separated by a thin glass capillary. In the no-choice test, insects were exposed to only one kind of food—treated or control. Dishes were kept in the rearing chamber at 29 ± 1 °C in the dark for three days. After this period, the remaining uneaten oat flakes were reweighed, and the average weight of food was calculated. The amount of eaten food was the basis for calculating the deterrence coefficients (relative R and absolute A). Three coefficients of deterrence were calculated: (1) Relative, R = [C − E/C + E] × 100 (in the choice test) (2) Absolute, A = [CC − EE/CC + EE] × 100 (in the no-choice test) (3) Total, T = A + R, where C and CC is the amount of food consumed from control discs; E and EE is the amount of consumed food from discs treated with tested compounds [13].

The value of total coefficients of deterrence (T) ranges from −200 to +200. The negative value indicates that a compound is a feeding attractant. The positive value indicates feeding deterrent activity, within the range 0–50 described as week, 50–100—medium, 100–150 good and 150–200—very good.

### 3.12. Statistical Analysis

The mean values of the deterrence coefficients were compared by means of one-way analysis of variance (ANOVA) followed by Tukey’s tests at a level of *P* < 0.05.

## 4. Conclusions

Reported for the first time *A. ochraceus*-catalyzed hydrolysis of racemic lactones is a useful tool to obtain both enantiomerically enriched (*S*)-γ-ethylidene-γ-lactones (−)-**2a**–**d** and (+)-(*R*)-γ-ketoacids **3a**–**d**. All eight compounds obtained by the biotransformation were not reported previously in optically active forms and three γ-ketoacids **3b**,**d** were not described previously in the literature. Enantioselectivity of the transformation reaction is strictly related to a size of aryl substituent. Most of lactones **2a**–**d** are stronger feeding deterrents for adults than for larvae of the lesser mealworm, whereas ketoacids **3a**–**d** reduce feeding of larvae to a higher extent. The influence of a size of aryl substituent on antifeedant activity is also significant, especially the presence of *p*-methoxy group at the phenyl ring significantly increase the activity. No clear differences between racemic and enantiomerically enriched forms of lactones were observed. The exception was lactone **2a**; in this case (*S*)-enantiomer is more active against larvae whereas (*R*)-enantiomer is more active against adults of the lesser mealworm. The racemic lactones with methylsubstituted benzene ring (**2b**) and 1,3-benzodioxole system (**2d**), due to their high activity against both developmental stages, can find practical applications for *A. diaperinus* control.

## Figures and Tables

**Table 1:** Conversions and enantiomeric excesses of unreacted (−)-(*S*)-isomers obtained after hydrolysis of lactones (±)-**2a**–**d** by *Aspergillus ochraceus* AM370.

Substrate				
	2a	2b	2c	2d
Conversion ^a^ [%]	48	55	52	48
*ee* [%]	77	56	22	15

^a^ According to GC.

**Table 2:** Feeding deterrent activity of (±)-γ-ethylidene-γ-lactones **2a**–**d **and products of their biotransformations: (−)-(*S*,*E*)-**2a**–**d** and (+)-(*R*)-**3a**–**d** against lesser mealworm *Alphitobius diaperinus*.

Entry	Compound	Deterrence Coefficients ^1^					
		Larvae			Adults		
		A	R	T	A	R	T
1	(±)-**2a**	8.59 ± 5.63a	64.01 ± 6.21ab	72.60 ± 12.43a	64.13 ± 11.44bc	83.07 ± 3.60cd	147.19 ± 14.07def
2	(±)-**2b**	84.4 ± 4.49d	78.96 ± 2.25bc	163.36 ± 4.66b	81.16 ± 10.67de	72.54 ± 5.25abcd	153.7 ± 6.68def
3	(±)-**2c**	53.87 ± 6.71d	90.48 ± 4.61bc	144.35 ± 5.82ab	91.88 ± 1.97e	93.64 ± 0.73d	185.52 ± 2.09ef
4	(±)-**2d**	67.92 ± 13.34d	96.19 ± 0.58c	164.11 ± 11.88b	71.12 ± 9.97de	85.56 ± 6.81bcd	156.67 ± 8.73def
5	(−)-(*S*)-**2a**	9.33 ± 1.34a	94.87 ± 1.52bc	104.20 ± 3.04a	22.47 ± 8.98a	90.83 ± 3.58cd	113.31 ± 12.53abc
6	(−)-(*S*)-**2b**	30.15 ± 4.16ab	70.62 ± 12.86ab	100.76 ± 12.76a	47.19 ± 3.48ab	90.23 ± 2.86cd	137.41 ± 6.85bcd
7	(−)-(*S*)-**2c**	55.11 ± 12.11bcd	85.84 ± 2.21bc	140.95 ± 10.88b	94.69 ± 1.64e	94.06 ± 1.22d	188.75 ± 1.87f
8	(−)-(*S*)-**2d**	44.10 ± 2.07bc	91.49 ± 2.19bc	135.59 ± 1.33ab	76.07 ± 9.66de	94.29 ± 1.39d	170.36 ± 9.85def
9	(+)-(*R*)-**3a**	43.61 ± 3.38bc	86.87 ± 3.78bc	130.48 ± 5.33a	33.12 ± 3.65ab	75.59 ± 4.38bcd	108.71 ± 7.34abc
10	(+)-(*R*)-**3b**	82.60 ± 0.39d	66.45 ± 7.29ab	149.05 ± 6.32b	29.18 ± 2.19a	65.17 ± 5.62ab	94.35 ± 5.92ab
11	(+)-(*R*)-**3c**	59.08 ± 1.43d	50.08 ± 5.99ab	109.16 ± 2.14a	30.67 ± 4.48ab	53.16 ± 2.93a	83.83 ± 5.61a
12	(+)-(*R*)-**3d**	60.16 ± 6.23bcd	39.59 ± 9.08a	99.75 ± 7.51a	31.36 ± 5.99ab	50.22 ± 11.46a	81.58 ± 9.25a
13	Azadirachtin [42]	97.00	82.62	179.62	99.14	98.44	197.58

^1 ^Values are the means of the four replicates, each set up with ten larvae or adults (*n *= 40). A: absolute coefficients; R: relative coefficients; T: total coefficients. Means followed by the same letters within each column are not significantly different (one-way ANOVA and Tukey’s test (*P < *0.05).
